# Pre-Hospital Delays Represent Unnoticed Intervals That Affect Mortality Rates in Geriatric Hip Fractures: A Prospective Cohort Study

**DOI:** 10.7759/cureus.44773

**Published:** 2023-09-06

**Authors:** Akash K Ghosh, Sandeep Patel, Devendra Chouhan, Tanvir Samra, Rajendra K Kanojia, Ashish Bhalla

**Affiliations:** 1 Department of Orthopaedics, Postgraduate Institute of Medical Education and Research, Chandigarh, IND; 2 Department of Anesthesia and Intensive Care, Postgraduate Institute of Medical Education and Research, Chandigarh, IND; 3 Department of Internal Medicine, Postgraduate Institute of Medical Education and Research, Chandigarh, IND

**Keywords:** early surgery, hip fracture, neck of femur, intertrochanteric fracture, geriatric

## Abstract

Introduction

Surgery is recommended within 24-48 hours for geriatric hip fractures. In developing countries. However, delayed presentation to the hospital due to various factors often precludes surgery from occurring within these recommended intervals. Therefore, our objective was to identify the hurdles that prevent early surgery for geriatric hip fractures and assess their effect on mortality.

Methods

A prospective cohort study was conducted with 78 geriatric patients (age > 60 years) who suffered hip fractures between September 2019 and November 2020. The demographic, American Society of Anesthesiologists (ASA) classification, Charlson Comorbidity Index (CCI), injury to admission, admission to surgery, and injury to surgery time were all recorded for each patient. A follow-up was conducted at one month and six months postoperatively for each patient. Mortality rate at 30 days and causes for delay in presentation to the hospital and delayed surgery were assessed. Multivariate logistic regression was done to assess the risk factors for 30-day mortality.

Results

The mean age of the patients was 74.2 years, and 64.1% of the patients were female. The mean (SD) injury-to-admission time was 3.45 (5.50) days, and the admission-to-surgery time was 4.28 (3.03) days. A total of 41% of patients had delayed presentation, commonly due to a lack of local healthcare infrastructure, financial constraints, and a lack of care providers. Furthermore, 65.3% of the patients underwent delayed surgery, and 44% faced organizational delays. Thus, the 30-day mortality rate was calculated at 19.2%, while the six-month mortality rate was 25.6%. The injury to admission time (OR 1.22 [1.03-1.44; p = 0.018]) and CCI were found to be risk factors in the 30-day mortality (OR 1.76 [0.93-3.33; p = 0.085]).

Conclusions

Pre-hospital delays and CCI are risk factors for short-term mortality following hip fractures. This underlines the need to generate awareness, improve the referral chain, and establish protocol-based care in hospitals. Further studies are required to assess the socioeconomic factors involved in the delayed treatment of geriatric hip fractures in developing countries.

## Introduction

Hip fractures are a leading cause of mortality and morbidity worldwide in the geriatric population. Moreover, due to the aging population and increasing life expectancy, the burden of hip fractures is increasing, with one-third of the population of India expected to be in the geriatric age group by the year 2050 [1]. The mortality rate due to hip fractures is estimated to be 5%-10% after one month and 12%-27% a year after surgery [2].

In developed countries, geriatric hip fractures are considered a surgical emergency; thus, protocols are in place for these patients to be operated on as early as possible [3]. Most studies recommend that the patients undergo surgery within 24 to 48 hours of the injury occurring [4,5]. However, conflicting evidence has been presented on whether early surgery reduces mortality, complication rate, length of hospital stay, and overall cost of care [6-8].

Furthermore, the current target of performing surgery within 48 hours of the injury is not regularly replicable in developing countries, such as India, due to sociocultural factors, the lack of knowledge, and the basic infrastructure available at the grassroots [9]. Hence, in developing countries, the injury to admission time has emerged as an additional time interval that deserves serious consideration. Therefore, the aim of this study was to identify the reasons for the surgical delays from the onset of the injury and to study the effect of these delays on the overall mortality rate. We hypothesized that delays in surgery would lead to greater mortality in these patients.

## Materials and methods

This study obtained permission from the institutional ethics committee (Ref: NK/5711/MS/911). Then, a prospective observational study was performed from September 2019 to November 2020 and consecutively included all the patients above 60 years of age who had presented with isolated, closed, proximal femur fractures (neck of femur, intertrochanteric, subtrochanteric) to a tertiary care center in northern India. Patients under 60 years were excluded from the study, as were patients with pathological or multiple fractures and patients who possessed other associated injuries.

At presentation, the baseline characteristics of each patient, such as age, gender, ASA grade, comorbidities, CCI (Charlson Comorbidity Index), cognitive status (with a short portable mental status questionnaire), preinjury functional status (ambulatory with or without aid, or non-ambulatory), pain status (as per the verbal rating scale for pain), and date and time of injury, were recorded. If the time that the injury occurred until the presentation to our hospital was more than 48 hours, then the cause for the delay was recorded and categorized as either lack of knowledge, lack of local healthcare infrastructure, lack of care provider, or financial constraint. 

A multidisciplinary team comprising an orthopedic surgeon, a geriatrician, and an anesthetist reviewed each patient and established the diagnosis, treatment plan, and any further investigations that were required for optimization or surgical clearance. The time of each assessment was recorded, and the investigation and time from admission to surgery were also noted. The mode of anesthesia and the type of surgery (proximal femoral nailing, bipolar hemiarthroplasty, or total hip replacement) were also recorded. If the patient underwent surgery later than 48 hours after admission, the reason for the delay was documented and classified as patient-related medical factors, medical organizational delays, or surgical organizational delays. Postoperatively, the length of hospital stays, cognitive status (as per the short portable mental status questionnaire [SPMSQ]), pain (as per the VRS), and day of postoperative mobilization were noted. Mortality and cause of death were assessed at 30 days and six months from the date of the surgery. Similarly, functional scores were noted at 30 days (social dependence score and mobility score) and at six months (Harris hip score and SF-12 questionnaire). Patient follow-ups were performed telephonically at 30 days and through an outpatient clinic at six months. 

All the collected data were tabulated in MS Excel, and the statistical analysis was performed using IBM Corp. Released 2020. IBM SPSS Statistics for Windows, Version 27.0. Armonk, NY: IBM Corp. Data are reported as mean (SD) or median as appropriate for continuous variables and as percentages (%) for categorical variables. A Shapiro-Wilk test was performed to determine the normal distribution of the data. The means of variables were compared to identify any significant association and/or correlation using parametric and non-parametric tests, such as the t-test, Mann-Whitney test, Chi-squared test, and Fisher’s exact test, while the Pearson correlation test was used for correlation. 

Then, all the variables with a significant association were analyzed using univariate linear regression for continuous variables and logistic regression for categorical variables, where the 30-day mortality was taken as a dependent variable. Next, the variables showing significance in the univariate regression (p > 0.2) were selected by ‘bidirectional stepwise selection’ and a multivariate regression analysis was conducted using the same dependent variables. Then, a ROC curve was plotted to determine the predictive value of the significant risk factors from the multivariate regression analysis.

## Results

**Figure 1:**
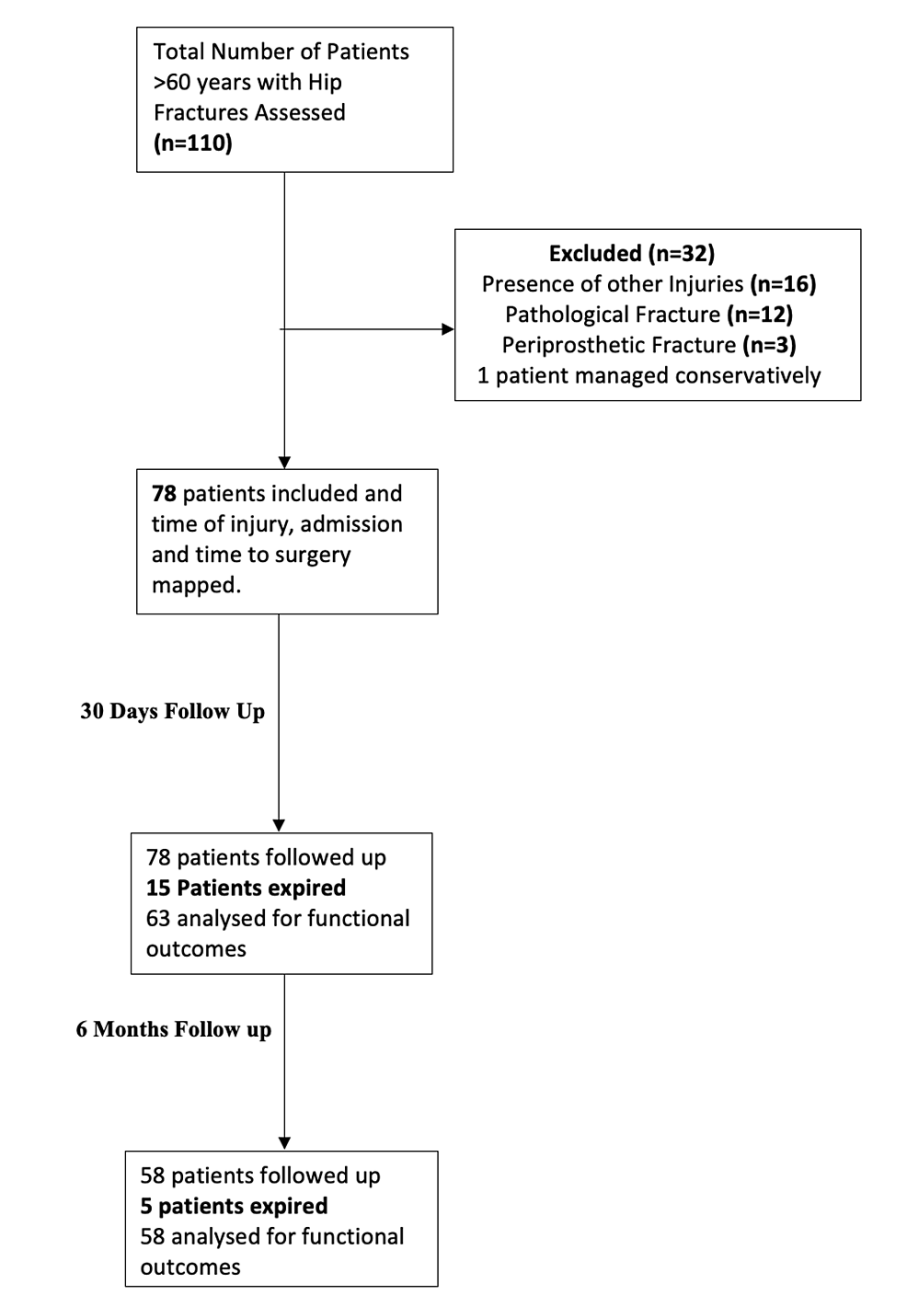
Patient flow in the study

A total of 78 patients were included in this study from September 2019 to November 2020 (Figure 1), with ages ranging from 60 to 110 years and a mean age of 74.27 (9.29) years. There were 28 males (36%) and 50 females (64%) (Table 1). Intertrochanteric fractures were the most common injury (67.9%), followed by neck femur fractures (29.5%) and subtrochanteric femur fractures (2.6%). Most of the patients were categorized as ASA Grade 2 (48.7%) and ASA Grade 3 (38.5%). Moreover, 88.5% of the patients were suffering from at least one comorbidity, with hypertension (64.1%) and diabetes (34.6%) representing the most common comorbidities. The mean (SD) CCI value was 4.54 (1.38), while 65.4% of the patients were independently ambulatory, 28.2% ambulated with the help of a walking aid, and the remaining 6.4% were non-ambulatory before the injury.

**Table 1:** Demographic details of study population (n=78)

Parameters	Mean ± SD, Frequency (%)
Age (Years)	74.27 ± 9.79
Age	
51-60 years	5 (6.4%)
61-70 years	26 (33.3%)
71-80 years	26 (33.3%)
81-90 years	18 (23.1%)
>90 years	3 (3.8%)
Gender	
Male	28 (35.9%)
Female	50 (64.1%)
Pre-Injury Functional Status	
Ambulatory	51 (65.4%)
Ambulatory with walking aid	22 (28.2%)
Non-Ambulatory	5 (6.4%)
ASA	
Grade 1	6 (7.7%)
Grade 2	38 (48.7%)
Grade 3	30 (38.5%)
Grade 4	4 (5.1%)
CCI	4.54 ±1.38
Type of Fracture	
Intertrochanteric	53 (67.9%)
Neck of Femur	23 (29.5%)
Subtrochanteric	2 (2.6%)
Length of Hospital Stay (Days)	9.69 ± 5.01

Overall, 49.3% of the patients underwent bipolar hemiarthroplasty, 46.7% of the patients underwent proximal femoral nailing, and 4% of the patients received a total hip replacement. Additionally, 80% of the patients were operated on under combined spinal and epidural anesthesia. Postoperatively, half of the patients were relocated to an orthopedic ward, 44.9% were transferred to a high-dependency unit, and 5.1% were moved to the intensive care unit. The mean (SD) length of hospital stay was 9.69 (5.01) days, with stays ranging from 3 to 39 days.

The mean (SD) injury to admission time was 3.45 (5.50) days, with a range of 0 to 27 days, and the time from admission to surgery was 4.28 (3.03) days, with a range of 1 to 20 days. The mean (SD) injury-to-surgery time was 7.43 (5.68) days.

A total of 41% of the patients presented to the hospital more than 48 hours after the injury. The reasons cited for the delays were a lack of local healthcare infrastructure (15.3%), a lack of care providers (10.3%), financial constraints (9%), and a lack of knowledge (6.4%) (Table 2). Financial constraints caused the longest mean (SD) delay to the admission of 11.57 (7.66) days. Males (4.9 days) had a greater mean injury-to-admission time compared to females (2.98 days) (p > 0.05). The non-ambulatory patients had a greater mean injury-to-admission time (8.8 days) compared to the ambulatory patients (3.45 days) (p > 0.05).

**Table 2:** Reasons for delay in presentation (n = 78) and reasons for delay in surgery (n = 75)

Reason for delay	Frequency (%)
Reason for Delay in Presentation	
No delay	46 (59.0%)
Lack of Local Healthcare Infrastructure	12 (15.4%)
Lack of Care Provider	8 (10.3%)
Financial Constraints	7 (9.0%)
Lack of Knowledge	5 (6.4%)
Reason for Delay in Surgery	
No Delay	26 (34.7%)
Medical Organization	18 (24.0%)
Medical Cause	16 (21.3%)
Surgical Organization	15 (20.0%)

Furthermore, 34.7% of patients were operated on within 48 hours of admittance, 33.3% were operated on within 48-96 hours of admission, and 32% underwent a delay of more than 96 hours before surgery. Patient-related medical factors accounted for 21.3% of these extensive delays and included reasons such as their conditions requiring optimization, including psychiatric exacerbation, anemia, and the need for dialysis.

Medical organizational causes resulted in 24% of the delays, including delayed clearances and waiting for cardiac stress thallium testing, while 20% were the result of surgical organizational delays, such as a lack of OT, the “weekend effect”, and financial issues that affected the availability of the required implants.

Age (OR 0.08 (95% CI: 0.01-0.15; p = 0.028), female gender (r2 1.27 (0.20-2.73; p = 0.034), and CCI (r2 0.49 [0.01-0.98; p = 0.05]) were factors associated with the time from admission to surgery. When the variables were adjusted for each other using the multivariate regression analysis, only the CCI (r 2 0.61 (0.09-1.13; p = 0.021) was found to affect the time from admission to surgery. Additionally, using the ROC analysis, the CCI >/ = 6 was also found to be predictive of surgical delay, whereas the injury to admission time had no correlation with the admission to surgery time.

The mortality rate was 19.2% at 30 days and 25.6% at six months. Unfortunately, three patients died before surgery; however, they were included in the study in the ‘intention to treat analysis’. Cardiac (40%) and renal (20%) abnormalities were the most common causes of mortality. Age (OR 1.06 [95% CI: 0.99-1.13; p = 0.081]), being female (RR 1.12; 95% CI: 0.45-2.93), ASA Grades 3 and 4 (p = 0.043), CCI (OR 1.59 (95% CI: 1.00-2.64; p = 0.050), and general anesthesia (RR 4; 95% CI: 1.5-10.12) were found to be associated with higher rates of mortality (Table 3). When the risk factors for 30-day mortality were assessed using a multivariate logistic regression, only injury to admission time (OR 1.22 [1.03-1.44; p = 0.018]) and CCI (OR 1.76 [0.93-3.33; p = 0.085]) were found to be risk factors for mortality. In the ROC analysis, CCI >/ = 4 was found to be a significant predictor of mortality. There were no statistically significant differences in mortality based on injury to surgery times or admission to surgery times. 

**Table 3:** Regression analysis for factors affecting 30-day mortality

Dependent: 30 days mortality		No	Yes	OR (univariable)	OR (multivariable)
Age (Years)	Mean (SD)	73.3 (10.3)	79.2 (5.2)	1.06 (0.99-1.13, p=0.081)	1.01 (0.88-1.15, p=0.933)
Gender	Male	23 (88.5)	3 (11.5)	-	-
	Female	40 (83.3)	8 (16.7)	1.53 (0.40-7.53, p=0.556)	0.43 (0.03-5.82, p=0.509)
Injury to Admission Interval (Days)	Mean (SD)	2.9 (4.8)	4.4 (5.5)	1.05 (0.93-1.17, p=0.380)	1.22 (1.03-1.44, p=0.018)
Pre-Injury Functional Status	Ambulatory	46 (92.0)	4 (8.0)	-	-
	Ambulatory With Walking Aid	14 (70.0)	6 (30.0)	4.93 (1.24-21.74, p=0.025)	8.45 (0.58-193.23, p=0.134)
	Non-Ambulatory	3 (75.0)	1 (25.0)	3.83 (0.17-39.65, p=0.289)	2.03 (0.01-194.17, p=0.763)
Charlson Comorbidity Index	Mean (SD)	4.4 (1.4)	5.3 (1.3)	1.59 (1.00-2.64, p=0.055)	1.76 (0.93-3.33, p=0.085)
Admission to Surgery Interval (Days)	Mean (SD)	4.3 (3.2)	3.9 (1.6)	0.95 (0.70-1.16, p=0.659)	0.77 (0.37-1.20, p=0.372)

Moreover, there were no variables that were significantly associated with the injury-to-surgery times. However, patients with longer injury-to-surgery times remained in the hospital for longer durations (correlation coefficient [rho] = 0.38; p < 0.001). Neither a delay in surgery nor delayed admission caused any statistically significant difference in the functional outcomes of patients at 30 days (social dependence and mobility scores) and six months (SF-12 and Harris hip score) after surgery. The post hoc statistical power of the study was calculated to be 36.2%, with 30-day mortality as the outcome measure and assuming alpha to be 0.05.

## Discussion

The importance of early surgery for geriatric hip fractures continues to be a topic of debate, with most developed countries possessing guidelines recommending that surgery occur within 24-48 hours of the injury. Moreover, in developed countries, the time taken from admittance to surgery is used as a surrogate for injury to surgery time [10] owing to the robust health infrastructure and referral systems that are currently in place, meaning that there are negligible delays from injury to admission. However, the scenario is very different in developing countries, such as India, where the injury to admission times and admission to surgery times need to be treated at two different intervals with their own sets of time lags. To the best of our knowledge, our study is the first to analyze these intervals separately and assess the causes of delay and their impact on mortality. 

The mean (SD) injury-to-admission time in our study was 3.45 (5.50) days and ranged from a few hours to 27 days, with 41% of the patients presenting to clinical care later than 48 hours after the injury occurred. The delays in admission in this study are similar to those found in other Indian studies; for example, Dash et al. reported a mean (SD) injury to admission time of 18 (16.9) days, with 85% of patients presenting later than 24 hours [11]. Kulshreshtha et al. also found that more than 50% of the patients were admitted later than 48 hours after injury, with the mean time from injury to admission being 1.7 days, with a range of up to 14 days [12]. Further, in an observational study by Rath et al., the authors noticed that 52% of the patients presented later than 24 hours after injury [13]. Similarly, in a retrospective cohort study from Brazil, Vidal et al. report a mean injury-to-admission time of 3.1 days, with 50% of the patients presenting later than 48 hours [14].

In stark contrast, the HIP ATTACK trial reported a median injury-to-admission time of three hours, with the upper limit in both the accelerated and standard care groups being 15 hours [15]. In a prospective cohort study conducted by Orosz et al. in New York, USA, only 17% of the patients presented to the hospital later than 24 hours after sustaining an injury [16]. A few other Western studies even excluded patients who presented later than 48 hours [17]. Thus, the scenario in developing nations differs vastly from the one in developed nations, where immediate or early presentation to the hospital after an injury is the norm; thus, the injury to surgery time and admission to surgery time are considered to be the same.

The most common cause of delay that was offered in this study was a lack of local healthcare infrastructure (15.4%), followed by a lack of care providers (10.3%), financial constraints (9.0%), and a lack of knowledge of the condition (6.4%). The patients with financial constraints had the longest mean admission to surgery time (11.57 ± 7.66 days). Thus, the lack of universal insurance coverage, coupled with the prevalence of poverty in India and the high healthcare costs associated with hip fractures, make financial constraints a significant barrier to early surgery. 

These findings were similar to other studies in India, whereby Dash et al. (2014), in a prospective multicentric study, reported that patients had to travel a mean distance of 86.4 kilometers to access quality healthcare [11]. Additionally, the common causes for delay were found to be the ignorance of family members and misguiding quacks and nursing home practices at the grassroots level, which delayed the time taken for a referral; therefore, patients often spent so much at the first point of care that they faced financial constraints in purchasing implants when treated at the tertiary center [11]. 

Within the hospital, medical causes (patient factors) caused the maximum delay to surgery (mean [SD] = 6.31 [4.21] days), followed by medical organizational delays (mean [SD] = 6.11 [2.83] days), and surgical organizational delays (mean [SD] = 3.93 [0.88] days). Amongst medical causes, most patients were delayed due to delirium, followed by patients requiring preoperative dialysis due to AKI or CKD. A few patients were delayed owing to a requirement for preoperative blood transfusions due to anemia, while other causes were exacerbations of COPD and CHF. Thirteen patients in our study were delayed due to the stress thallium test, while the other cause for medical organizational interruption was delayed medical clearances, which were either due to delayed consultation or the physician not attending to the patient on time. The surgical organizational delays were due to the lack of immediate availability of an operating theater, the "weekend effect", wherein the patients who were admitted on a weekend experienced a longer wait for surgery due to reduced staffing, and some patients were delayed due to financial constraints in procuring implants.

Dash et al. reported a similar admission to surgery time (mean [SD] = 3.7 [2.9] days; range 1-12 days), whereby only 26.8% of patients underwent surgery within 48 hours of admission, with similar reasons being cited for the delays, including public holidays, financial constraints, and fixed days for surgeons [11]. Kulshreshtha et al. reported a mean time of 1.8 days with a maximum of 19 days [12]. In the study by Rath et al., 70% of patients underwent surgery later than 48 hours after admission [13]. Vidal et al. reported a mean time of 13 days from admission to surgery [14]. 

The HIP ATTACK trial reported a median time from admission to surgery of six hours in the accelerated care group and 24 hours in the standard care group [15]. In a prospective cohort study of 1234 patients in Spain, Lizaur-Utrilla et al. (2018) observed that the mean time to surgery was 4.1 days in the medical delays group (23.2% of patients) and 3.9 days in the organizational delays group (27.5% of patients) [8]. The most common reasons for the medical delays were antiplatelet reversals and cardiac arrythmias, while the common reasons for the organizational delays were unavailable operating rooms, delayed medical clearances, and the ”weekend effect” [8]. However, the study did not specify whether the time observed was from injury to surgery or from hospital admission to surgery.

The causes of in-hospital delays are similar worldwide and were the most modifiable factors in our study. The availability of greater resources over the weekend, universal insurance coverage, appropriate cardiac screening, and multidisciplinary care to ensure quicker referrals and clearances can seemingly address most of the prevailing issues. There is also the need to change perceptions of hip fractures, similar to a myocardial infarction or a stroke, to evoke a greater urgency in their management. 

In our study, the 30-day mortality rate was 18.9%, and the six-month mortality rate was 25.6%. The mortality rate was higher than in other contemporary studies, whereby Kulshreshtha et al. reported a 7.7% 1-year mortality rate and Dhibar et al. published a 12.3% mortality rate at three months and 16.7% mortality at six months [12,18]. The HIP ATTACK trial reported a similar in-hospital mortality rate of 10%, compared to 11.3% in this study [15]. In a systematic review by Abrahamsen et al. (2009), the 30-day mortality was found to be 13.3%, and the six-month mortality was 15.8%, which increased to 24.5% at one year and was 34.5% at two years [19]. The higher mortality rate could be due to the study center being used as the referral center, thereby resulting in a higher proportion of unoptimized patients or patients who could not be treated at a smaller center, meaning they were referred to the hospital. 

Expectedly, almost all studies reported age as a significant factor that affected mortality. In our study, mortality was highest in octogenarians (33.3%), followed by patients in the 71-s80 age group (30.8%). Moreover, the patients who died had a higher ASA grade, whereby patients in ASA Grade 4 had the highest mortality rate (75%), followed by Grade 3 (26.7%), which is also in agreement with previous studies. An increase in the ASA grade causes increases in anesthetic and postoperative complications and has also been shown to be associated with increased one-year mortality [20,21]. 

The CCI was found to be a risk factor in the 30-day mortality rate (OR 1.59; 95% CI: [1.00-2.64]; p = 0.050). Indeed, Cher et al., in their retrospective study, also found that CCI independently predicted both the short-term and long-term mortality rates in patients with geriatric hip fractures [22]. Hasan et al. also reported that an increase in the CCI predicted increased mortality and postoperative complications [23].

To our knowledge, this study represents the first study to identify injury-to-admission interval as an independent risk factor in early postoperative mortality. This interval assumes even greater significance in developing countries and should be the focus of further research into health planning and management.

In our study, the in-hospital delays and the overall injury to surgery time did not significantly affect mortality or functional outcomes. The absence of any significant difference in mortality between the patients who faced in-hospital delays and those who did not also signifies the importance of optimizing a geriatric patient immediately before surgery to avoid any additional organizational delays.

Our study has limitations. First, we had a small sample size compared to other similar studies. Therefore, continuing this study with more patients may yield more significant differences. Second, we calculated the time to admission and the time to surgery in days instead of hours, which most of the previous studies assessing the delay in surgery used as the unit of time since calculating time in hours would improve the accuracy of the study. Third, the surgical delays were specifically grouped into patient factors or organizational delays, meaning that adjustments were not made for combinations of delays. Finally, the use of specific medications pre-admission was not documented, and it was also not recorded whether the comorbidities were known before admission or diagnosed after the patient arrived at the hospital.

## Conclusions

Pre-hospital delay and CCI are risk factors for short-term mortality following hip fractures. This time interval is unique to developing and underdeveloped nations and is seldom addressed. This underlines the need to generate awareness, improve the referral chain, and set up protocol-based care in hospitals to reduce the delay in presentation to the hospital. Further studies are required to assess the socio-economics of delayed treatment of geriatric hip fractures in developing countries.
